# The Feasibility of High-Intensity Interval Training in Patients with Intensive Care Unit-Acquired Weakness Syndrome Following Long-Term Invasive Ventilation

**DOI:** 10.1186/s40798-021-00299-6

**Published:** 2021-02-01

**Authors:** Simon Wernhart, Jürgen Hedderich, Svenja Wunderlich, Kunigunde Schauerte, Eberhard Weihe, Dominic Dellweg, Karsten Siemon

**Affiliations:** 1 Department of Cardiology, Fachkrankenhaus Kloster Grafschaft, Annostrasse 1, 57392 Schmallenberg, Germany; 2 Department of Cardiology and Vascular Medicine, West German Heart- and Vascular Center, University Hospital Essen, University Duisburg-Essen, Hufelandstrasse 55, 45147 Essen, Germany; 3 Medistat-Biomedical Statistics, Medistat GmbH, Kronshagen, 24119 Germany; 4 Department of Pneumology, Fachkrankenhaus Kloster Grafschaft, Schmallenberg, 57392 Germany; 5 Institute of Anatomy and Cell Biology of the Philipps-University Marburg, Marburg, 35037 Germany

**Keywords:** ICUAWS, MCT vs. HIIT in critically ill patients, Early rehabilitation facility

## Abstract

**Background:**

Intensive care unit-acquired weakness syndrome (ICUAWS) can be a consequence of long-term mechanical ventilation. Despite recommendations of early patient mobilisation, little is known about the feasibility, safety and benefit of interval training in early rehabilitation facilities (ERF) after long-term invasive ventilation.

**Methods and Results:**

We retrospectively analysed two established training protocols of bicycle ergometry in ERF patients after long-term (> 7 days) invasive ventilation (*n* = 46). Patients conducted moderate continuous (MCT, *n* = 24, mean age 70.3 ± 10.1 years) or high-intensity interval training (HIIT, *n* = 22, mean age 63.6 ± 12.6 years). The intensity of training was monitored with the BORG CR10 scale (intense phases ≥ 7/10 and moderate phases ≤ 4/10 points). The primary outcome was improvement (∆-values) of six-minute-walk-test (6 MWT), while the secondary outcomes were improvement of vital capacity (VC_max_), forced expiratory volume in 1 s (FEV_1_), maximal inspiratory pressure (PI_max_) and functional capabilities (functional independence assessment measure, FIM/FAM and Barthel scores) after 3 weeks of training. No adverse events were observed. There was a trend towards a greater improvement of 6 MWT in HIIT than MCT (159.5 ± 64.9 m vs. 120.4 ± 60.4 m; *p* = .057), despite more days of invasive ventilation (39.6 ± 16.8 days vs. 26.8 ± 16.2 days; *p* = .009). VC_max_ (∆0.5l ± 0.6 vs. ∆0.5l ± 0.3; *p* = .462), FEV_1_ (∆0.2l ± 0.3 vs. ∆0.3l ± 0.2; *p* = .218) PI_max_ (∆0.8 ± 1.1 kPa vs. ∆0.7 ± 1.3pts; *p* = .918) and functional status (FIM/FAM: ∆29.0 ± 14.8pts vs. ∆30.9 ± 16.0pts; *p* = .707; Barthel: ∆28.9 ± 16.0 pts vs. ∆25.0 ± 10.5pts; *p* = .341) improved in HIIT and MCT.

**Conclusions:**

We demonstrate the feasibility and safety of HIIT in the early rehabilitation of ICUAWS patients. Larger trials are necessary to find adequate dosage of HIIT in ICUAWS patients.

**Supplementary Information:**

The online version contains supplementary material available at 10.1186/s40798-021-00299-6.

## Key Points

- High-intensity interval training (HIIT) is feasible in critically ill patients with intensive care-acquired weakness syndrome (ICUAWS) after long-term invasive ventilation.
- HIIT is safe in ICUAWS patients after long-term invasive ventilation.
- Six minute-walk-test (6 MWT) improves in both high-intensity and moderate continuous training groups.

## Background

A major problem after long-term intensive care treatment is the intensive care unit-acquired weakness syndrome (ICUAWS), which can be triggered by sepsis, multiorgan failure, glucocorticoids, neuromuscular blocking agents, hyperglycemia and immobility itself [1]. Reported incidence rates of ICUAWS range from 25 to 100% and may independently contribute to the one year mortality rate [2]. The underlying pathophysiology has not been entirely elucidated and the diagnosis remains the domain of a meticulous neurological exam supported by neurophysiological measurements [2]. Patients may represent with myopathic or neuropathic symptoms or a combination of both.

Physical activity (PA) reduces morbidity and mortality by reducing the burden of arrhythmias and improvement of cardiorespiratory fitness (CRF) through modulating lipid profiles, increasing aerobic capacities in adolescents, grown-ups and the elderly [3–5]. High-intensity interval (HIIT) and moderate continuous training (MCT) are established methods to preserve and boost endurance in professional and recreational sports as well as in patients [6–8]. Several studies have also demonstrated improvements in aerobic capacity (VO_2peak_) as well as cardiac remodelling in heart failure patients with reduced ejection fraction (HF_r_EF) treated with HIIT [9]. Furthermore, HIIT seems to improve left- and right-ventricular contractile function [10], and reduces right-ventricular hypertrophy [11] and the burden of depression in heart failure patients [12]. It may also unfold extra-cardiac effects, for instance by altering DNA methylation to improve retinal microvasculature [13], or may alleviate endothelial dysfunction [14].

The first multicenter-randomised trial comparing HIIT (training between 90 and 95% of maximal heart rate, HR_max_) and MCT in HFrEF patients did not show differential effects on left-ventricular end-diastolic diameter (LVEDD) or VO_2peak_ [15]. Although there was improvement in both groups, this effect was not maintained after 1 year or a discontinuation of training. Importantly, no significant difference in serious adverse events was observed between the groups in this high-risk population [15]. HIIT seems to be as safe as other training modalities, even in high-risk patients, and compliance may even be higher due to reduced exercise time and less monotonous workouts [16]. Moreover, recent randomised trial data suggest even lower all-cause mortality in older patients performing HIIT [17].

Early mobilisation seems promising in the recovery of critically ill patients [18–21]. The early adaptation of training has recently been implemented in intensive care units (ICUs) by using bed cycle ergometry [22] and electrical muscle stimulation [23]. Since rehabilitation from ICUAWS has been shown to be prolonged, early recognition and initiation of physical rehabilitation measures is warranted as it may decrease its incidence and improve patients’ functional status [24, 25]. A randomised trial has been launched to investigate the impact of different physiotherapeutic interventions in ICU patients (FITonICU, German Clinical Trial Register, identifier DRKS00010269) [26]. However, evidence is scarce on the timing and dosage of training in critically ill patients with ICUAWS transferred from the ICU to early rehabilitation facilities (ERF) [27]. To our knowledge, no data exists on HIIT in early rehabilitation following prolonged weaning from invasive ventilation. We aimed to fill this gap by examining the feasibility of HIIT in these patients.

We hypothesised that HIIT leads to a more pronounced increase in six-minute-walk-test (6 MWT) than MCT after a training period of three weeks (primary outcome). As a secondary outcome, we analysed between-group differences between functional indices (Barthel and FIM/FAM, functional independence/assessment measure), lung function parameters (VC_max_: maximal vital capacity; FEV_1_: forced expiratory ventilation after one second; KCO: carbon monoxide transfer coefficient) and inspiratory muscle function (maximal inspiratory pressure at 0.1 s, P0.1; maximal inspiratory pressure, PI_max_; and respiratory capacity, P0.1/PI_max_). By providing a feasibility study on HIIT in critically ill patients, we aim to set the stage for a multi-center, prospective, randomised trial to investigate mortality.

## Methods

### Study Design

We conducted a retrospective, non-randomised trial comparing two established training protocols (MCT and HIIT) of our early rehabilitation facility (ERF). All eligible patients who trained in our ERF between 01.02.2019 and 29.02.2020 were retrospectively analysed following ethical approval (Ethics Committee University Münster, Germany, 2020-418-f-S). Insurance covers 3 weeks of ERF training in our country, including physical training on 5 days per week. During the studied period, 162 patients were admitted with 66 completing all 3 weeks of training. Twenty patients did not meet the inclusion criteria, resulting in 46 patients who were entered into the study (24 in the MCT and 22 in the HIIT groups). Group allocation was done on the day of ERF admission by shared-decision making of the attending physician and physiotherapist based on the patients’ estimated physical and cognitive capabilities to perform training of varying intensities. Patients suffering from severe ICUAWS unable to increase workload at ERF admission were allocated to the MCT group, the others to the HIIT group.

### Participants

We included patients referred to our ERF directly from the adjacent intensive care-unit following long-term invasive mechanical ventilation (defined as at least 7 days of invasive ventilation via an endotracheal tube or a tracheostoma) of any cause. All patients had developed ICUAWS, which was diagnosed by an independent consulting neurologist just before admission to the ERF according to current recommendations [2, 28]. Patients’ physical and cognitive eligibility for inclusion in the study were determined in a team decision among the ward’s physiotherapists, nurses and physicians. Inclusion criteria were prolonged weaning (> 7 days of invasive ventilation) from the respirator, and a RASS (Richmond Agitation Sedation Scale) of zero points. In order to participate, patients had to be extubated; a persistent tracheostoma, or the necessity for non-invasive ventilation were no exclusion criteria. A modified Rankin scale (mRS) of no more than three points was required in order to perform testing and training in case of a history of stroke [29]. Exclusion criteria were acute infection and sepsis, acute heart failure, respiratory failure (defined as pH < 7.25 and CO_2_ > 70 mmHg), persistent hypotension (mean arterial pressure, MAP < 65 mmHg) with a need for iv treatment with inotropic drugs, refractory hypertension (defined as a systolic blood pressure > 150 mmHg despite triple antihypertensive therapy), known uncontrolled endocrinological diseases and a neurological impairment too severe to take part in testing and training, defined as a mRS of more than three points.

### Interventions

Bicycle ergometry training (MOTOmed viva2) in both groups was undertaken 5 days per week for 3 weeks (15 endurance sessions, one session per day); no supervised training took part on the two remaining days of the week (5 days of consecutive training followed by 2 days of rest). The MCT group performed 14 min of training, comprising warm-up and cool-down phases (each two minutes) at 0–1/10 points and 10 min of moderate intensity ≤ 4/10 points (total workout time 14 min) on a modified BORG scale (BORG CR10; ranging from 0 to 10, 0 being no and 10 being maximal exertion), which correlates with quantitative performance measures [30–32].

**Fig. 1:**
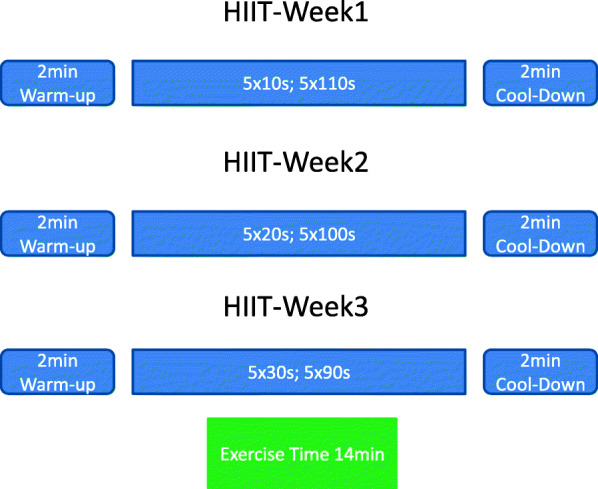
Increased workload during the HIIT (high-intensity interval training) cycles (10–30 s) over the training period of three weeks. *s* seconds

Training in the HIIT group was defined as five cycles, each comprising a period at a workload ≥ 7/10 and an intertwined cool-down phase ≤ 4/10 points (total workout time 14 min). The percentage of high-intensity training was increased over time (Fig. 1). The run-in phase aimed to acquaint patients with the training method. Compared to other HIIT studies [33], a shorter interval was chosen to avoid rapid lactate accumulation [34], which would have detrimental effects in ICUAWS patients. Active recovery was chosen in the HIIT group, because it facilitates lactate clearance [35]. Maximal cardiopulmonary exercise testing and lactate measurements to determine VO_2peak_, ventilatory thresholds were not feasible. Baseline maximal exercise testing to determine HR _peak_ was not performed due to safety concerns immediately following ICU discharge.

Prior to each session, patients were asked whether they felt able to take part in the exercise procedure, and clinical capability was assessed by both a physiotherapist and a physician. Blood pressure (RR) was measured before training and immediately after exercise termination. During exercise, peripheral oxygen saturation (S_p_O_2_) and heart rate (HR) were continuously monitored. After each full minute during exercise, patients had to rate their intensity on the BORG CR10 scale. Prior to exercise, capillary blood gas analysis was performed. A pH < 7.25, paCO_2_ > 70 mmHg and a paO_2_ < 60 mmHg (with or without oxygen supply) were exclusion criteria for participation in the session.

Criteria for premature exercise termination were angina, exertional dyspnea, a heart rate > 180/min (as an arbitrary, but clinically reasonable upper limit in critically ill patients), a drop of S_p_O_2_ < 80% and an unwillingness of the patient to continue. Oxygen was applied during exercise whenever necessary to maintain a minimal S_p_O_2_ ≥ 85%.

Background proprioceptive and resistance training were performed five times per week (15 sessions in total) in both groups for a period of 30 min. Resistance training contained machine-guided activiation of the major muscle groups of the upper and lower limbs in three series per muscle group and 20 repetitions per series at low intensity. Physiotherapists supervised correct movement execution. Proprioceptive training was performed with balance pads at the start of each day’s training session for 5 min.

In both groups, patients received daily swallowing therapy for persistent dysphagia. In case of insufficient coughing reflexes, cough assist devices and inhalation training were applied, and bronchoscopies were conducted in case of the failure of conservative methods to expectorate the mucus. Training sessions on the application of long-term oxygen therapy (LTOT) and non-invasive ventilation (NIV) were provided by our personnel until patients were able to use the devices independently.

### Assessments

Baseline assessment on the day of ERF admission from the ICU comprised a physical exam, laboratory testing, blood gas analysis, transthoracic echocardiography (TTE), abdominal sonography and venous ultrasound to exclude lower-limb thrombosis. The sonographic exams were conducted by an experienced physician. Left-ventricular ejection fraction (LVEF) on TTE was determined with the eyeballing method, while right-ventricular function was assessed by measuring tricuspid annular plane systolic excursion (TAPSE), whereby relevant valvular dysfunction was defined as at least grade II stenosis or insufficiency according to current guidelines [36, 37].

In order to assess exertion and intensity of training patients were instructed to use a modified BORG scale (BORG CR10; ranging from 0 to 10, 0 being no and 10 being maximal exertion), which correlates with quantitative performance measures [30–32]. Motivation to take part in the sessions was documented prior to and after exercise. Values were reported on an adapted numeric rating scale (NRS, 0 being no motivation at all, 10 being fully motivated), which is commonly used as a tool to measure pain and is easily applicable [38].

Walking ability and estimation of cardiorespiratory fitness were assessed at baseline (*t*_0_) and after three weeks (*t*_1_) by using 6 MWT which was conducted according to an established protocol [39]. Patients were seated for 10 min on a chair next to the starting line prior to testing. The supervisor walked behind the tested person to ensure safety and prevent falling.

In order to analyze improvement of respiratory muscle dysfunction as a result of ICUAWS, lung function testing (VC_max_, FEV_1_, KCO) and inspiratory muscle force (P0.1, PI_max_, P0.1/PI_max_) were calculated at both time points. As a measure of independence and functionality we used the Barthel and combined FIM/FAM (30-item test, ranging from 30 to 210 points, with 210 points illustrating a patient’s functional independence) scores at *t*_0_ and *t*_1_. The test was performed in a quiet atmosphere by a specialised nurse together with the patient according the established protocol [40].

### Statistical Methods

SPSS (IBM Corp. Released 2016. IBM SPSS Statistics for Windows, Version 24.0. Armonk, NY: IBM Corp.) was used for exploratory and descriptive data analyses. The Kolmogorov-Smirnov test (including Lilliefors significance correction) and Shapiro-Wilk test were applied for normality testing. Comparability of groups for nominally-scaled factors was analysed with the χ^2^-test (Fisher’s exact test) and for ratio-scaled variables with the non-paired, two-sided *t* test and *U* test (Mann-Whitney), where appropriate. The *U* test was also applied as a confirmation test despite the presence of a normal distribution (normality testing would only reveal greater deviations from a normal distribution in small samples). Thus, only significance in both *t* and *U* testing can be regarded as reliable to demonstrate ‘real’ differences between the groups. In order to consider the covariable duration of invasive ventilation, ANCOVA was used to test between-group differences on 6 MWT. Regarding the explorative intention of this study, the significance level was set at α = .05 without adjusting for multiple testing. In order to estimate sample size for future randomised controlled trials, power calculation for the primary outcome was undertaken using Cohen’s *d* (supplement 1).

Error bar plots (mean ± standard error of mean) were used to illustrate the development of HR, S_p_O_2_ and motivation throughout the 15 days of exercise. Post-hoc analysis of longitudinal data was analysed by two-factorial analyses of variances (repeated measures ANOVA) to evaluate the effects of training, time, and possible interactions between them. Sphericity was tested by Mauchly’s *W* statistic and the degrees of freedom were adjusted by Greenhouse-Geisser epsilon. The significance level was set at α = 0.05.

## Results

We analysed a total of 46 patients, 24 in the MCT and 22 in the HIIT group. Reasons for prior long-term invasive ventilation in the HIIT group were pneumonia (*n* = 11, 50.0%), COPD exacerbation (*n* = 7, 31.8%), peritonitis (*n* = 2, 9.1%), acute bypass surgery (*n* = 1, 4.6%) and acute heart failure (*n* = 1, 4.6%). In the MCT group, invasive ventilation was induced by pneumonia (*n* = 13, 54.2%), COPD exacerbation (*n* = 7, 29.2%), acute heart failure (*n* = 3, 12.5%) and acute gastrointestinal bleeding (*n* = 1, 4.1%). Tracheostomy had to be performed in 86.4% (*n* = 19) of HIIT and 83.3% (*n* = 20) of MCT patients. Further, 70.8% (*n* = 17) in the MCT and 59.1% (*n* = 13) in the HIIT group were male (*p* = .538). The groups did not differ in the occurrence of hypothyroidism (MCT: 79.2%, *n* = 19; HIIT: 81.8%, *n* = 18; *p* = .990), polyneuropathy (MCT: 91.7%, *n* = 22; HIIT: 90.9%, *n* = 20; *p* = .990) or a history of long-term steroid use (MCT: 83.3%, *n* = 20; HIIT: 72.7%, *n* = 16; *p* = .484).

**Fig. 2:**
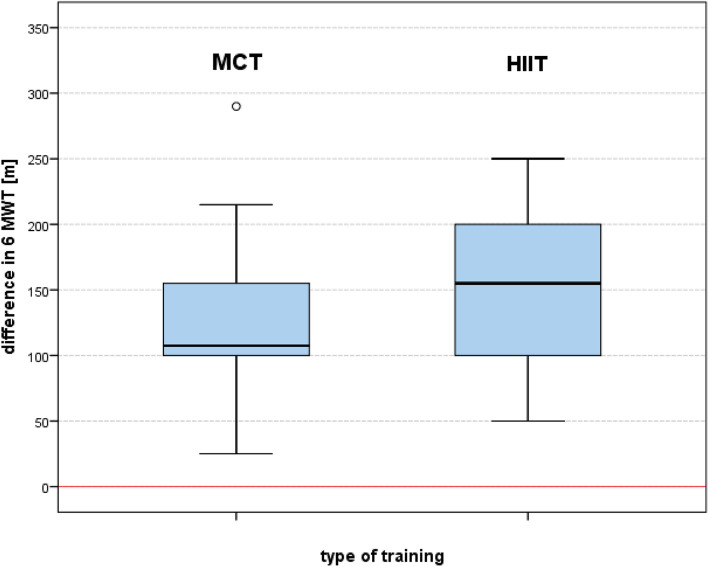
Differences of increase in six-minute walk test [m] between MCT (moderate continuous training) and HIIT (high-intensity interval training) after three weeks of training (box-whisker-plot)

The HIIT group consisted of significantly more hypertensive, diabetic and heart failure patients (Table 1) and was significantly longer under invasive ventilation (Table 2). Training intervention led to a marked increase of 6 MWT in both groups (∆*t* in MCT 120.4 ± 60.4 m vs. 159.5 ± 64.9 m in HIIT), with a trend towards higher 6 MWT gain in the HIIT group compared to the MCT group: After confirmation of normality, *t* testing revealed a significant absolute difference between the groups (*p* = .041), Mann-Whitney testing failed to confirm this (*p* = .057 for absolute differences, *p* = .882 for *t*_0_ and *p* = .059 for *t*_1_, see Table 3 and Fig. 2). The *duration of invasive ventilation* as a covariable did not have a significant influence on the difference of 6 MWT (*p* = .949), and there was no significant group effect (*p* = .055) in ANCOVA analysis.

**Table 1:** Baseline characteristics

Variable	MCT (*n* = 24)	HIIT (*n* = 22)	*p* value
Hypertension			
Yes	4.2% (*n* = 1)	27.3% (*n* = 6)	*p* = .043*
No	95.8% (*n* = 23)	72.7% (*n* = 16)	
Afib			
Yes	41.7% (*n* = 10)	50.0% (*n* = 11)	*p* = .768
No	58.3% (*n* = 14)	50.0% (*n* = 11)	
Oral anticoagulation			
Yes	29.2% (*n* = 7)	45.5% (*n* = 10)	*p* = .361
No	70.8% (*n* = 17)	54.5% (*n* = 12)	
Statin use			
Yes	70.8% (*n* = 17)	81.8% (*n* = 18)	*p* = .497
No	29.2% (*n* = 7)	18.2% (*n* = 4)	
Diabetes			
Yes	50.0% (*n* = 12)	81.8% (*n* = 18)	*p* = .032*
No	50.0% (*n* = 12)	18.2% (*n* = 4)	
BMI > 30 kg/m^2^			
Yes	41.7% (*n* = 10)	63.6% (*n* = 14)	*p* = .155
No	58.3% (*n* = 14)	36.4% (*n* = 8)	
Nicotine			
Yes	12.5% (*n* = 3)	22.7% (*n* = 5)	*p* = .451
No	87.5% (*n* = 21)	77.3% (*n* = 17)	
Stroke			
Yes	62.5% (*n* = 15)	86.4% (*n* = 19)	*p* = .096
No	37.5% (*n* = 9)	13.6% (*n* = 3)	
Chronic heart failure			
Yes	25.0% (*n* = 6)	63.6% (*n* = 14)	*p* = .016*
No	75.0% (*n* = 18)	36.4% (*n* = 8)	
Coronary artery disease			
Yes	79.2% (*n* = 19)	81.8% (*n* = 18)	*p* = 0.990
No	20.8% (*n* = 5)	18.2% (*n* = 4)	
COPD			
Yes	54.2% (*n* = 13)	54.5% (*n* = 12)	*p* = .990
No	45.8% (*n* = 11)	45.5% (*n* = 10)	
LTOT			
Yes	41.7% (*n* = 10)	59.1% (*n* = 13)	*p* = .376
No	58.3% (*n* = 14)	40.9% (*n* = 9)	
NIV			
Yes	54.2% (*n* = 13)	54.5% (*n* = 12)	*p* = .990
No	45.8% (*n* = 11)	45.5% (*n* = 10)	
LVEF			
> 50%	62.5% (*n* = 15)	90.8% (*n* = 20)	*p* = .261
40–50%	25.0% (*n* = 6)	4.6% (*n* = 1)	
< 40%	12.5% (*n* = 3)	4.6% (*n* = 1)	
Valvular dysfunction			
Yes	75.0% (*n* = 18)	81.8% (*n* = 18)	*p* = .725
No	25.0% (*n* = 6)	18.2% (*n* = 4)	

Baseline characteristics of patients in the moderate continuous (MCT, *n* = 24) and high-intensity interval training (HIIT, *n* = 22) groups. *Afib* Atrial fibrillation, *BMI* Body mass index [kg/m^2^]. Stroke: this illustrates a history of stroke with a current modified Rankin scale of no more than three points. *COPD* Chronic obstructive lung disease, *LTOT* Long-term oxygen treatment, *NIV* Non-invasive ventilation, *LVEF* Left-ventricular ejection fraction (categorised with eyeballing in transthoracic echocardiography). Valvular dysfunction was defined as at least grade II stenosis or insufficiency on transthoracic echocardiography. Mean values ± standard deviations are depicted. Significance is denoted with an asterisk (*p* < .05). Differences were assessed with chi-squared/Fisher’s test

**Table 2:** Comparison of baseline variables between groups

Baseline variable	MCT (*n* = 24)	HIIT (*n* = 22)	*p* value
Age [years]	70.3 ± 10.1 years	63.6 ± 12.6 years	*p* = .143
Duration of invasive ventilation [days]	26.8 ± 16.2 days	39.6 ± 16.8 days	*p* = .009*
sPAP [mmHg]	36.9 ± 7.9 mmHg	35.3 ± 6.2 mmHg	*p* = .487
TAPSE [mm]	19.5 ± 4.0 mmHg	20.7 ± 5.0 mmHg	*p* = .596
Haemoglobin [g/dl]	10.9 ± 1.4 g/dl	10.7 ± 1.2 g/dl	*p* = .857
NTproBNP [pg/ml]	3376.0 ± 5367.2 pg/ml	2407.6 ± 5119.0 pg/ml	*p* = .699
Cystatin C clearance [ml/min]	47.4 ± 20.1 ml/min	52.7 ± 29.7 ml/min	*p* = .806
Folic acid [μg/l]	14.3 ± 7.6 μg/l	10.2 ± 4.0 μg/l	*p* = .070
B12 [ng/l]	512.0 ± 195.9 ng/l	449.8 ± 165.9 ng/l	*p* = .198
Vitamin D_3_ [μg/l]	21.4 ± 13.9 μg/l	22.7 ± 14.8 μg/l	*p* = .961

Comparison of baseline variables between study groups. MCT (*n* = 24): moderate continuous training. *HIIT* (*n* = 24) High-intensity interval training. *sPAP* [mmHg] Systolic pulmonary artery pressure on transthoracic echocardiography. *TAPSE* [mmHg] Tricuspid annular plane systolic excursion on transthoracic echocardiography. *NTproBNP* [pg/ml] N-terminal prohormone of brain natriuretic peptide. Mean values ± standard deviations are depicted. Significance is denoted with an asterisk (*p* < .05). Differences were assessed with *t* testing

**Table 3:** Gain in 6 MWT between MCT and HIIT

6min walk test [m]	MCT (*n* = 24)	HIIT (*n* = 22)	*p* value
∆*t*	120.4 ± 60.4 m	159.5 ± 64.9 m	*p* = .057
*t*_0_	32.7 ± 51.6 m	33.0 ± 47.2 m	*p* = .882
*t*_1_	153.1 ± 69.4 m	192.5 ± 76.0 m	*p* = .059

Absolute gain in six-minute-walk-test [6 MWT, m] (∆*t*, *t*_1_–*t*_0_, primary endpoint) as well as differences between *t*_0_ and *t*_1_ between *MCT* (Moderate continuous training) and *HIIT* (High-intensity interval training) after three weeks of training. Mean values ± standard deviations are depicted. Differences were assessed with Mann-Whitney and *t* testing

Analysis of secondary outcomes revealed consistent results between *t* and Mann-Whitney testing, with no significant differences between secondary outcomes (see Table 4). Premature exercise termination due to dizziness occurred on five occasions (two in the HIIT and three in the MCT group). Symptoms resolved within a few minutes. No major adverse events were observed.

**Table 4:** Differences in secondary endpoints between MCT and HIIT

Variable	MCT (*n* = 24)	HIIT (*n* = 22)	*p* value
Barthel index [pts]	∆*t*: 25.0 ± 10.5pts	∆*t*: 28.9 ± 16.0pts	*p* = .341
	*t*_0_: 27.5 ± 15.5pts	*t*_0_: 30.2 ± 12.0pts	*p* = .697
	*t*_1_: 52.5 ± 12.3pts	*t*_1_: 59.1 ± 17.7pts	*p* = .143
FIM + FAM [pts]	∆*t*: 30.9 ± 16.0pts	∆*t*: 29.0 ± 14.8pts	*p* = .707
	*t*_0_: 60.3 ± 17.4pts	*t*_0_: 69.2 ± 14.1pts	*p* = .190
	*t*_1_: 91.2 ± 18.7pts	*t*_1_: 98.1 ± 18.0pts	*p* = .240
VC_max_ [l]	∆*t*: 0.5l ± 0.3	∆*t*: 0.5l ± 0.6	*p* = .462
	*t*_0_: 1.7l ± 0.7	*t*_0_: 1.9l ± 0.7	*p* = .312
	*t*_1_: 2.2l ± 0.8	*t*_1_: 2.4l ± 0.9	*p* = .334
FEV_1_ [l]	∆*t*: 0.3l ± 0.2	∆*t*: 0.2l ± 0.3	*p* = .218
	*t*_0_: 1.2l ± 0.6	*t*_0_: 1.4l ± 0.7	*p* = .272
	*t*_1_: 1.5l ± 0.6	*t*_1_: 1.6l ± 0.7	*p* = .732
KCO [%]	∆*t*: 7.8 ± 8.2%	∆*t*: 4.6 ± 7.2%	*p* = .176
	*t*_0_: 55.7 ± 30.9%	*t*_0_: 57.5 ± 25.7%	*p* = .651
	*t*_1_: 63.5 ± 26.6%	*t*_1_: 62.1 ± 23.5%	*p* = .996
P0.1 [kPa]	∆*t*: 0.1 ± 0.1 kPa	∆*t*: 0.1 ± 0.1 kPa	*p* = .684
	*t*_0_: 0.2 ± 0.2 kPa	*t*_0_: 0.3 ± 0.2 kPa	*p* = .939
	*t*_1_: 0.3 ± 0.2 kPa	*t*_1_: 0.3 ± 0.2 kPa	*p* = .435
PI_max_ [kPa]	∆*t*: 0.7 ± 1.3 kPa	∆*t*: 0.8 ± 1.1 kPa	*p* = .918
	*t*_0_: 2.8 ± 1.4 kPa	*t*_0_: 3.4 ± 1.3 kPa	*p* = .214
	*t*_1_: 3.5 ± 1.6 kPa	*t*_1_: 4.2 ± 1.5 kPa	*p* = .171
P0.1/PI_max_ [%]	∆*t*: − 1.3% ± 7.3	∆*t*: − 0.3% ± 4.2	*p* = .331
	*t*_0_: 10.8% ± 8.6	*t*_0_: 8.9% ± 7.9	*p* = .310
	*t*_1_: 9.5% ± 5.8	*t*_1_: 8.6% ± 5.7	*p* = .609

Differences in secondary endpoints (∆*t*, *t*_1_–*t*_0_; *t*_0_ and *t*_1_) between MCT (moderate continuous training) and HIIT (high-intensity interval training) after three weeks of training. Illustration of Mann-Whitney testing. Mean values and standard deviations are depicted. Barthel index [pts, points]: score ranging from 0 to 100, 100 representing complete independence. FIM + FAM [pts, points]: functional independence/assessment measure (score ranging from 30 to 210, 210 illustrating no functional limitation). VC_max_ [l]: Maximal vital capacity. FEV_1_ [l]: forced expiratory volume in one second. KCO [%]: carbon monoxide transfer coefficient. P0.1 [kPa]: maximal inspiratory pressure at 0.1 s. PI_max_ [kPa]: maximal inspiratory pressure. P0.1/PI_max_ [%]: respiratory capacity. Mean values ± standard deviations are depicted. Differences were assessed with Mann-Whitney and *t* testing

**Fig. 3:**
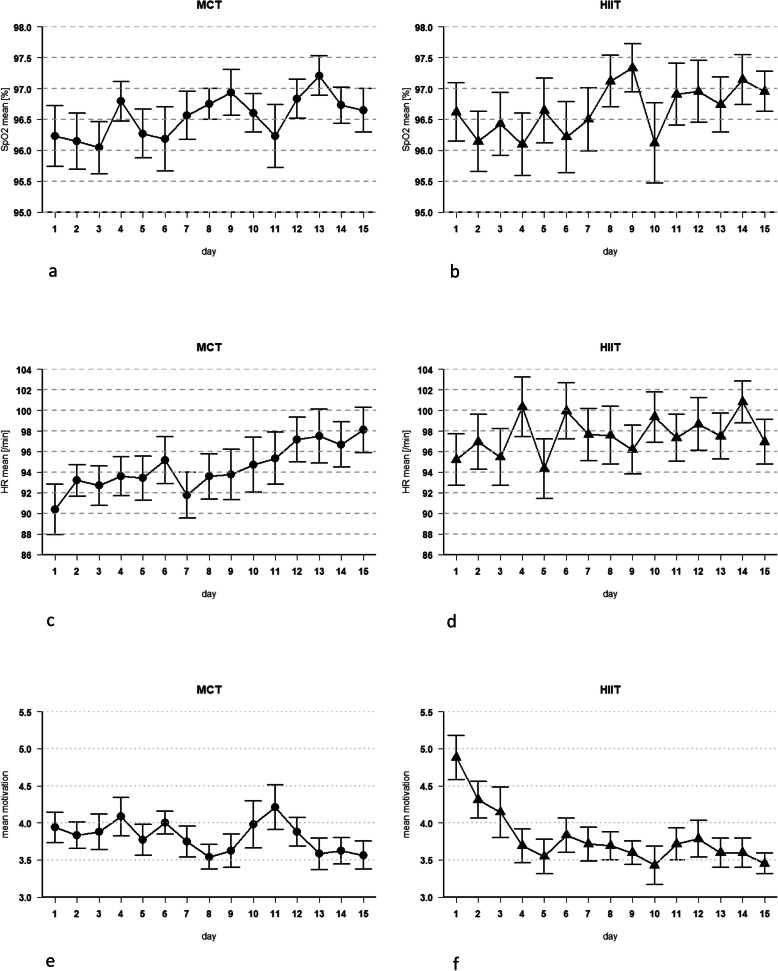
Mean values (± standard error) of MCT (moderate continuous training) and HIIT (high-intensity interval training) across the fifteen days of exercise. **a** S_p_O_2mean_ [%] in MCT. **b** S_p_O_2mean_ [%] in HIIT. **c** HR_mean_ [/min] in MCT. **d** HR_mean_ [/min] in HIIT. **e** Mean motivation in MCT [scale from 1 to 10, with 10 representing the highest level of motivation]. **f** Mean motivation in HIIT [numeric rating scale, NRS, from 1 to 10, with 10 representing the highest level of motivation]. Mean motivation is calculated from the motivation score prior to and post exercise

Over the training period of 3 weeks and fifteen sessions, we found a significant increase in S_p_O_2max_ (*p* = .008), HR_max_ (*p* = .002) and HR_mean_ (*p* = .013) over time, without a difference between the groups (S_p_O_2max_: *p* = .947; HR_max_: *p* = .420; HR_mean_: *p* = .233). A significant decrease in post-exercise motivation over time was documented (*p* < .001), without differences between the groups (*p* = .354). S_p_O_2min_ (*p* = .279; *p* = .596), S_p_O_2mean_ (*p* = .098; *p* = .768), HR_min_ (*p* = .207; *p* = .143) and pre- exercise motivation (*p* = .459; *p* = 257) did not differ over time or between the groups. Mean values are depicted in Fig. 3.

Though numerically higher in MCT, mean maximal workload (P_peak_, in Watt, W) did not differ at *t*_0_ (MCT 28.3 ± 10.1 W vs. HIIT 25.9 ± 10.5 W; *p* = .429) and *t*_1_ (MCT 49.6 ± 15.7 W vs. HIIT 49.5 ± 17.3 W; *p* = .994) between the groups. Both groups improved significantly between *t*_0_ and *t*_1_ (*p* < .001 each).

## Discussion

Our study is the first to provide evidence that interval training is feasible in patients after long-term invasive ventilation at a very early stage of rehabilitation. We retrospectively analysed training protocols that have been established in our ERF in patients with ICUAWS after long-term invasive ventilation over 3 weeks (15 days of exercise). Both groups increased their 6 MWT tremendously after 3 weeks, starting from very low values, with a trend towards better results in the HIIT group. We did not have a control group, which makes it impossible to clearly differentiate training effect from natural recovery. However, passive recovery without any intervention after long-term invasive ventilation would be unethical.

**Fig. 4:**
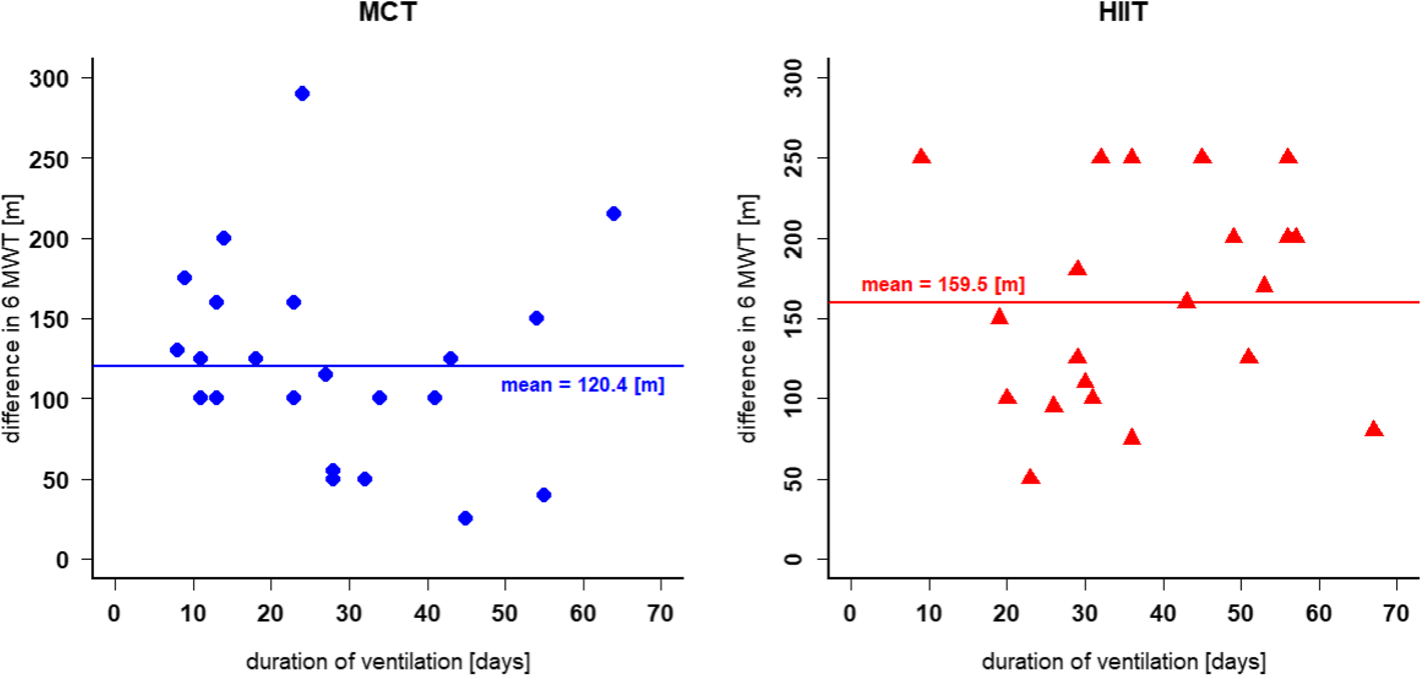
Improvement of 6 MWT [m] across duration of (invasive) ventilation [days] of each participant in the MCT (moderate continuous training, *n* = 24) and HIIT (high-intensity interval training, *n* = 22) group from *t*_0_ to *t*_1_. Mean improvements of the groups [m] are represented by the blue (MCT) and red (HIIT) horizontal lines

The HIIT group spent significantly more days on the ventilator (39.6 ± 16.8 days vs. 26.8 ± 16.2 days) at a slightly younger age, but still revealed a better absolute performance in 6 MWT increase after 3 weeks; duration of invasive ventilation did not have a significant influence on the primary outcome (see Fig. 4) and deviations of 6 MWT were quite large. We cannot claim that HIIT is superior to MCT in our population, because our study was under-powered to show such a difference (to detect a difference of 40 m in 6 MWT, a sample size of at least 40 patients per group would be necessary to achieve a power of 80%) (supplement 1). There is evidence that the duration of invasive ventilation is associated with the development of ICUAWS [41] and reduced long-term exercise performance [42]. In our study, all patients suffered from ICUAWS, but we did not find a significant association between the duration of invasive ventilation and improvement of 6 MWT. 6 MWT by itself is a predictor of survival in critically ill patients [43]. Due to our group allocation, patients with severe ICUAWS were more likely to receive MCT and tended to improve less than HIIT patients in terms of 6 MWT. We found that there were significantly more hypertensives (although low in number) and diabetics in the HIIT group, as well as more previously documented chronic heart failure cases. However, NTproBNP as a marker of heart failure was increased in both groups, which may be due to an ICU-acquired cardiomyopathy syndrome. Taken together, our data may cautiously suggest that severity of ICUAWS could be a predictor of poorer improvement of 6 MWT. A meticulous search for the presence and severity of ICUAWS on the ICU or ERF seems to be of paramount importance.

The lack of a difference in our primary study outcome (6 MWT) may be explained by several aspects: (1) training duration may have been too short, whereby extending our 3-week training programme to 6 or even 8 weeks may have led to more pronounced physiological adaptations; (2) subjective reporting of dyspnea—which led to the determination of intensity prescription—may not have met the required intensity level to achieve HIIT superiority (usually determined by either peak oxygen uptake,VO_2peak_, or maximal heart rate, HR_max_); and (3) the intensity of training after long-term invasive ventilation may not play a significant role. It might be more important to regularly engage in PA (of any intensity) under supervision of experienced personnel to improve CRF.

Although no significant between-group differences were detected in the secondary outcome, functional scores (Barthel and FIM/FAM), lung function testing and inspiratory muscle force markedly increased in HIIT and MCT after 3 weeks. This goes along with studies showing a beneficial effect of early and short-term ICU rehabilitation on inspiratory muscle capacity, lung function and functionality [21, 44, 45], both after cardiological [46–48] and pneumological [49, 50] events. However, a Cochrane review has pointed out that insufficient data exists on the long-term benefits of early rehabilitation in ICUAWS and the correct dosing of interventions is widely unknown [2, 45]. To date, no evidence of a survival benefit of early rehabilitation has been found in ICU patients. However, the heterogeneity of study protocols and patients’ history hampers generalisability: larger trials of early rehabilitation in ICUAWS patients have focused on the ICU setting (rather than specialised ERFs) with shorter times of invasive ventilation [51, 52]. Denehy et al. did not find a difference in 6 MWT in the intervention group after 12 months of follow-up after an ICU, ward-based and outpatient training [51]. However, this does not justify the assumption that there is no long-term effect of training in these high-risk patients; rather, there is a strong need for more trials analysing dosage and intensity of training, spanning ICU, ERF and outpatient care.

Similar to the SMARTEX heart failure study [15], in which many HIIT patients exercised below the prescribed intensity of > 90% of HR_peak_ (51%) and approximately 80% of MCT patients tended to work harder than prescribed, our HIIT group exercised at approximately 65–70% of HR_max_ on average if one uses the widely used formula *210- mean age in the HIIT group* (63.6 years). This is low considering other studies which also required mean exercise heart rate to be > 90% of HR_peak_ [17, 53]. However, it has to be noted that prescribing intensity at fixed % HR_peak_ may lead to considerable interindividual differences due to individual heart rate performance curves [54–56]. This may even become more of a problem in our ICUAWS groups with a relevant number (and different dosing) of beta-blockers, amiodarone or digitalis, which may further increase these differences. Additionally, chronotropic incompetence independent of drug effects may play a role in our population.

In our study, maintenance of exercise intensity was ensured by our physiotherapists, who accompanied every workout. However, mean P_peak_ was even lower in HIIT compared to MCT, which indicates that workload may not have been higher in the HIIT group. Heart rate as a means to guide exercise intensity, does not pay tribute to the metabolic demands of the muscle, which can only be expressed by measurement of lactate and calculation of lactate turn points in an incremental exercise test [54]. Tschakert and Hofmann have developed an elegant equation to calculate mean workload during HIIT by integrating peak and recovery workload as well as peak and recovery duration [34]. We would encourage future prospective studies on ICUAWS to use this equation following an incremental exercise test (preferably spiroergometry) to achieve comparable workloads among patients undergoing HIIT. Spiroergometry can be seen as a prerequisite to assess HR_peak_ and prescribe exercise intensities based on heart rate. The application of HR as a measure of intensity may also depend on the chosen HIIT protocol and may be problematic especially in protocols with short exercise phases. Furthermore, a reduction of bradycardic medication (as patients’ health improve) during the recovery process in ICUAWS patients may alter HR training corridors. Repetitive lactate measurements to depict the metabolic demands of ICUAWS patients may be more reliable to steer exercise intensity than HR itself.

We did not perform an incremental test in patients on the day of ERF admission (which was equivalent to ICU discharge) due to safety concerns. As we did not experience a single adverse event in our group of critically ill patients, this concern should no longer be upheld, provided the presence of personnel trained in medical emergency management. This is supported by the increasing data on the safety of HIIT [16, 57–59], even suggesting a reduced mortality in older patients compared to MCT [17]. Different workload durations ranging from 20 s to 4 min seem to be equally safe [15, 53, 60]. A recent meta-analysis found one major cardiovascular event in 17.000 HIIT training hours, which mainly occurred in the run-in phase [16]. For this reason, we chose to use a conservative run-in phase with a short duration of high-intensity workload during the first week in the HIIT group. As an exercise prescription for future studies on ICUAWS patients, we would recommend short bouts of higher intensity (< 1 min) to account for the underlying myopathy and higher metabolic challenges in these patients.

Interestingly, opposed to our clinical impression, we did not find a difference in training motivation in the two groups. Indeed, there was even a trend in the HIIT group to decline to the baseline of the MCT group over time. We measured the motivation of patients with the NRS score prior to and immediately after training. Asking patients about their motivation after recovery from training may have provided different results.

### Limitations

Our study has certain limitations. First, the BORG CR10 scale is a semi-quantitative yet validated method to measure workload [32]; however, objective criteria to measure intensity can only be applied by baseline exercise testing, which was not feasible directly after ICU discharge. Second, our population was heterogeneous in terms of baseline diseases (hypertension, diabetes and heart failure were more prominent in the HIIT group) and, as a retrospective study, was not stratified for diagnoses and medical treatments (best medical practice was applied in both groups). Third, our training protocol was arbitrary and illustrates a necessary compromise between training science to ensure maximal improvement in CRF and patient safety. Fourth, the significant increase of HR_mean_ and HR_max_ over time in both groups may be attributed to an increase in workload, indicating that intensity had also increased in the MCT group, a methodological problem which has also been observed in other studies [15, 17]. Also, mean P_peak_ did not differ between the groups, which may be due to the fact that the HIIT group was more severely impaired than the MCT group (more heart failure patients and longer periods of invasive ventilation), but may also indicate moderate rather than high-intensity interval training. Fifth, the training period was short and limited by the duration of insurance coverage of costs. Therefore, we cannot clearly differentiate the ‘natural recovery’ from actual training effects (a control group without bicycle ergometry would have been unethical in this phase of recovery). Spiroergometry with threshold determination and/or lactate measurements would have helped. Sixth, we did not have echocardiographic follow-up data available at the end of the study. Seventh, group allocation was done by shared decision making on the anticipated capabilities of patients, which led to a selection bias of clinically severe ICUAWS to the MCT rather than the HIIT group. Eighth, the diagnosis of ICUAWS by itself is difficult and was mainly dependent on the clinical diagnosis of an experienced neurologist. The history of a stroke itself was no exclusion criterion for the study, although an mRS of more than three points was deemed too much of a neurological impairment to take part in the study and was used as an exclusion criterion. However, we cannot entirely exclude a selection bias due to a different neurological status in the two groups as a result of the stroke (and not caused by the prolonged ventilation itself). Finally, both groups received the same amount of proprioceptive and strength training. However, a different physiological response in the HIIT and MCT group cannot be entirely excluded.

## Conclusion

We have demonstrated the feasibility and safety of HIIT in ERF patients with ICUAWS. HIIT patients displayed more days of invasive mechanical ventilation but still there was a trend towards greater 6 MWT improvements than in the MCT group after a training period of 3 weeks. Patients in both HIIT and MCT groups improved lung function, maximal inspiratory pressure and functional status after three weeks of structured ERF training. Intensity should be increased in the HIIT group to really implement high-intensity training. Prospective, randomised, multi-centre studies with sufficient power will be necessary to properly investigate whether HIIT is superior to MCT in ICUAWS patients: HIIT should be done following incremental (spiroergometric) exercise testing and determination of thresholds. Exercise prescription should be based on the formula by Tschakert and Hofmann to ensure workload consistency [34], aiming at high-intensity intervals (we would recommend 80–85%HR_max_) of less than 1-min duration to minimise muscular lactate accumulation. A run-in phase should be obligatory to ensure patients’ safety. Longer in- and out-of-hospital follow-ups will be necessary to investigate mortality in MCT and HIIT in ICUAWS patients.

## Supplementary Information

**Additional file 1.**

## Data Availability

The data underlying this article will be shared on reasonable request to the corresponding author.

## Abbreviations

ANOVA: Analysis of variance
BMI: Body mass index
COPD: Chronic obstructive pulmonary disease
CPET: Cardiopulmonary exercise testing
CRF: Cardiorespiratory fitness
ERF: Early rehabilitation facility
FEV_1_: Forced expiratory ventilation in 1 second
FIM/FAM: Functional independence measure/functional assessment measure
HF_r_EF: Heart failure with reduced ejection fraction
HIIT: High-intensity interval training
HR_min, max, mean_: Minimal, maximal, mean heart rate during exercise
ICU: Intensive care unit
ICUAWS: Intensive care unit-acquired weakness syndrome
KCO: Carbon monoxide transfer coefficient
LTOT: Long-term oxygen treatment
LVEDD: Left-ventricular end-diastolic diameter
LVEF: Left-ventricular ejection fraction
MCT: Moderate continuous training
mRS: Modified Rankin scale
NIV: Non-invasive ventilation
NRS: Numeric rating scale
NTproBNP: N-terminal prohormone of brain natriuretic peptide
PA: Physical activity
p_a_O_2_: Capillary partial pressure of oxygen
p_a_CO_2_: Capillary partial pressure of carbon dioxide
P0.1: Maximal inspiratory pressure at 0.1 seconds
P0.1/PI_max_: Respiratory capacity
PI_max_: Maximal inspiratory pressure
P_peak_: Performance at maximal power
RASS: Richmond agitation sedation scale
RR: Blood pressure
RVEF: Right-ventricular ejection fraction
6 MWT: Six-minute-walk-test
S_p_O_2min, max, mean_: Minimal, maximal and mean peripheral oxygen saturation
TAPSE: Tricuspid annular plane systolic excursion
TTE: Transthoracic echocardiography
VC_max_: Maximal vital capacity
VO_2peak_: Maximal oxygen uptake at peak performance
