# Supplementary material for: The Feasibility of High-Intensity Interval Training in Patients with Intensive Care Unit-Acquired Weakness Syndrome Following Long-Term Invasive Ventilation
Source: Sports Med Open. 2021 Feb 1;7:11. doi: 10.1186/s40798-021-00299-6 (PMC7849616; doi:10.1186/s40798-021-00299-6)
Supplement: Supplementary file 1 — Additional file 1. [file 40798_2021_299_MOESM1_ESM.docx]

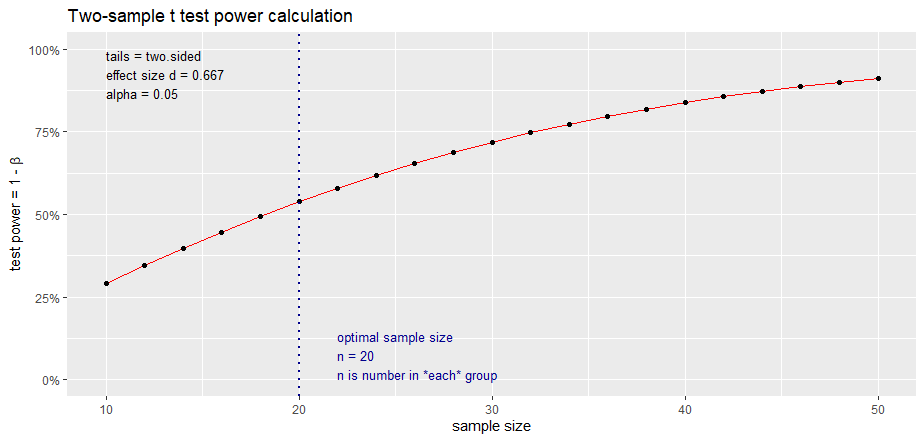


**Supplement 1.** Power calculation. Test power is illustrated across the sample size. Assuming a difference of 40m (±60m) for a sample size of n=20 (the term *optimal sample size* is defined by the statistical program and only refers to the employed sample size; a clinically optimal sample size will be considerably higher), an effect size of 0.667 is achieved using Cohen’s *d* based on the numerical between-group difference. By taking into consideration n=20; α=0.05, and an effect size of 0.667 the power for the two-sided t-test is 54%.
